# Supplementary material for: Single molecule secondary structure determination of proteins through infrared absorption nanospectroscopy
Source: Nat Commun. 2020 Jun 10;11:2945. doi: 10.1038/s41467-020-16728-1 (PMC7287102; doi:10.1038/s41467-020-16728-1)
Supplement: Supplementary file 1 — Supplementary Information [file 41467_2020_16728_MOESM1_ESM.pdf]

Supplementary Materials for

Single Molecule Secondary Structure Determination of Protein through Infrared Absorption Nanospectroscopy

Francesco Simone Ruggeri et al.

SUPPLEMENTARY NOTE 1

SHORT PULSE AND LOW POWER ORS-NANOIR

In our work, we aimed at unravelling the physical interaction between the gold probe, substrate and the soft biomolecules to develop a more accurate and sensitive AFM-IR mode (**Figure 1a,b**) to acquire IR absorption chemical spectra and maps at the single molecule nanoscale from soft biological samples, such as a single protein molecule.

We first studied the thermomechanical response of the cantilever on the bare gold substrate as a function of the power and the pulse width of the incident IR source (**Figure 1c,d**). Current commercially available AFM-IR systems typically exploit quantum cascade lasers (QCL) to use the resonance enhanced mode and increase the sensitivity of the measurement, with a typical power of the laser between 10-500 mW.[1] We found that when the tip is placed close by the gold substrate, already at very low laser power (~0.3 mW, ~0.1% of commercial IR sources) and pulse of 100 ns, the enhancement of the field can cause non-linearity and instability of the AFM-IR signal (**Supplementary Figure 3**). In order to reduce the instability, we added a mesh filter (product number 51-0507-01, 100 LPI, Industrial Netting, USA) in front of the laser exit to reduce the power on the sample of one order of magnitude. After the introduction of the mesh filter, the laser power varied between approximately 0.05-0.25 mW in the protein spectral range (**Supplementary Figure 1**). The mesh filter was placed in a fixed manner to simply reduce the power of the laser and access the linear and stable region of measurement. The laser power was hold constant as a function of laser pulse change. With 100 ns pulse width, we found a linear relationship between the laser power and the IR amplitude signal only below ~0.35 mW at 100 ns pulse width. Above this threshold the strong enhancement of the field causes non-linearity and instability (blue points, in **Figure 1d** and **Supplementary Figure 3**). Then, we measured the laser beam size (~60 μm) and we could calculate an intensity of illumination of $I = 4 \cdot 10^{-5}$ mW·μm^{-2}.

As the intensity of the field and of the IR amplitude signal are also determined by the duration of the laser pulse at each cycle of measurement of the thermomechanical response (pulse width in **Figure 1b**), we then studied the effect of the pulse width on the IR amplitude signal at a laser power of 0.2-0.22 mW. As now expected, we found that the IR amplitude signal increases linearly as a function of power until reaching a threshold of ~160 ns, causing again instability and non-linearity of the cantilever detection (**Figure 1d**).

We performed the characterisation of the linear regime of thermomechanical response by analysing the stability of the thermomechanical response at wavenumbers corresponding to the maximum of absorption of protein (1655 cm^{-1}) and substrate (1730 cm^{-1}), as well as to the maximum power of laser (**Supplementary Figure 3**). As a general approach to demonstrate

the stability of the signal, it is sufficient to observe the stability of the IR amplitude signal as a function of time, as shown in the **Supplementary Movies 1-2**. In these videos, we show that the IR amplitude is stable as a function of time at a power of 0.2 mW and a pulse width of 40 ns, while when the pulse width is increased to 200 ns, the signal is highly unstable; even if the saturation of the IR amplitude signal has not occurred. Thus, we can conclude that we measure in the linear and stable regime within all our spectral range, independently from the wavelength of interest.

Then, we studied the thermomechanical response on the protein and the substrate as a function of the frequency of the pulsed laser. The frequency contact resonance is the frequency of oscillation of the sample and cantilever in contact. Since the stiffness of the cantilever is fixed, a lower contact resonance corresponds to a softer sample, while a higher contact resonance to a stiffer sample. The IR amplitude can be maximised if the frequency of the pulse of the IR laser is matched with the mechanical contact resonance frequency between the sample and the tip by selecting a laser pulse frequency leading to the maximum of the IR amplitude signal (black dashed line in **Figure 1e**).[2] In **Figure 1e**, we show the IR amplitude as a function of the frequency of the pulse of the IR laser, at a laser power of 0.22 mW and at 1655 cm^{-1} (amide band I of protein) when the tip is placed on the top of a single protein (blue curve) and on the top of the bare gold substrate (orange curve). The contact resonance curve of the stiff gold appears at higher frequency of the softer protein. We start from the observation that the two IR amplitude curves on the protein and on the gold substrate are partially superimposed, thus the signal arising from the cantilever and the substrate significantly contributes to the signal measured for the protein. This effect demonstrates that when measuring an object with a diameter smaller than the typical diameter of the AFM tip (~60 nm), the cantilever is also excited by the probe and substrate, an effect which diminishes the sensitivity to the sample itself. To quantify this effect, we defined the signal-to-noise ratio on a single protein as the ratio of the signal on the protein and that on the gold (protein/gold signal). Then, we studied the protein/gold signal as a function of the selected contact resonance frequency (off-resonance, res_{off}) from the frequency corresponding to the maximal IR amplitude. We did not find a monotonic trend (**Figure 1f**), but instead that the maximal protein/gold signal increases linearly when measuring few kHz at lower frequency than the contact resonance frequency maximum, corresponding to the maximum of the IR amplitude (red dashed line in **Figure 1e**). Then, as expected, the signal to noise ratio decreases when measuring too far from the maximum of the contact resonance. The maximum protein/gold signal is found at a res_{off} ~ - 1-2 kHz (**Figure 1f**).

SUPPLEMENTARY NOTE 2

PROTEIN SPECTRA AND SECONDARY STRUCTURE QUANTIFICATION

The amide band I of thyroglobulin showed an amide band I peaked at about 1660 cm^{-1}, but with an additional shoulder at approximately 1640 cm^{-1}. The analysis of the AFM-IR spectra by the deconvolution of the amide I band, by second derivatives and the integration of the structural contribution in the amide I band (**Supplementary Figure 11**) shows now three major peaks corresponding to intramolecular β-sheets (31±5%), α-helix and random coil (54±5%), and β-turns (15±5%), which is again in excellent agreement with bulk quantification by FTIR (**Figure 4g**), to CD (**Supplementary Figure 4**) and previous results in literature by bulk approaches.[3] The spectrum of apoferritin exhibited a sharp amide band I centred at approximately 1660 cm^{-1} and only a major component at the same frequency in the second derivative. The deconvolution of amide band I and the integration of the area of the convolving structural contributions confirmed that the protein is characterised by an extensive α-helix secondary structure (81±5%), plus residual contributions from random coils and β-turns, which is in excellent agreement with the bulk FTIR (**Figure 4f**) and CD measurements we performed (**Supplementary Figure 3**), as well as we the know structure of the proteins.[4]

The major difference between the spectra was observed in the ratio between the Amide II band intensity, which was anyway not used for structural determination. Even if we normalised the spectra by the QCL emission profile (**Supplementary Figure 1**), the smaller value of the Amide II mode is related to the low laser power in this region. The laser power in the Amide II is three times lower than in the Amide I, as well as the Amide II is absorbing IR light approximately twice less than the Amide I, thus the detection of Amide II is at the limit of instrumental sensitivity. While, it has been recently reported that for ordered biomaterials the lower absolute value of this band could be attributed to polarisation effects,[5] we believe polarisation effects are not likely for isotropic proteins.

SUPPLEMENTARY FIGURES

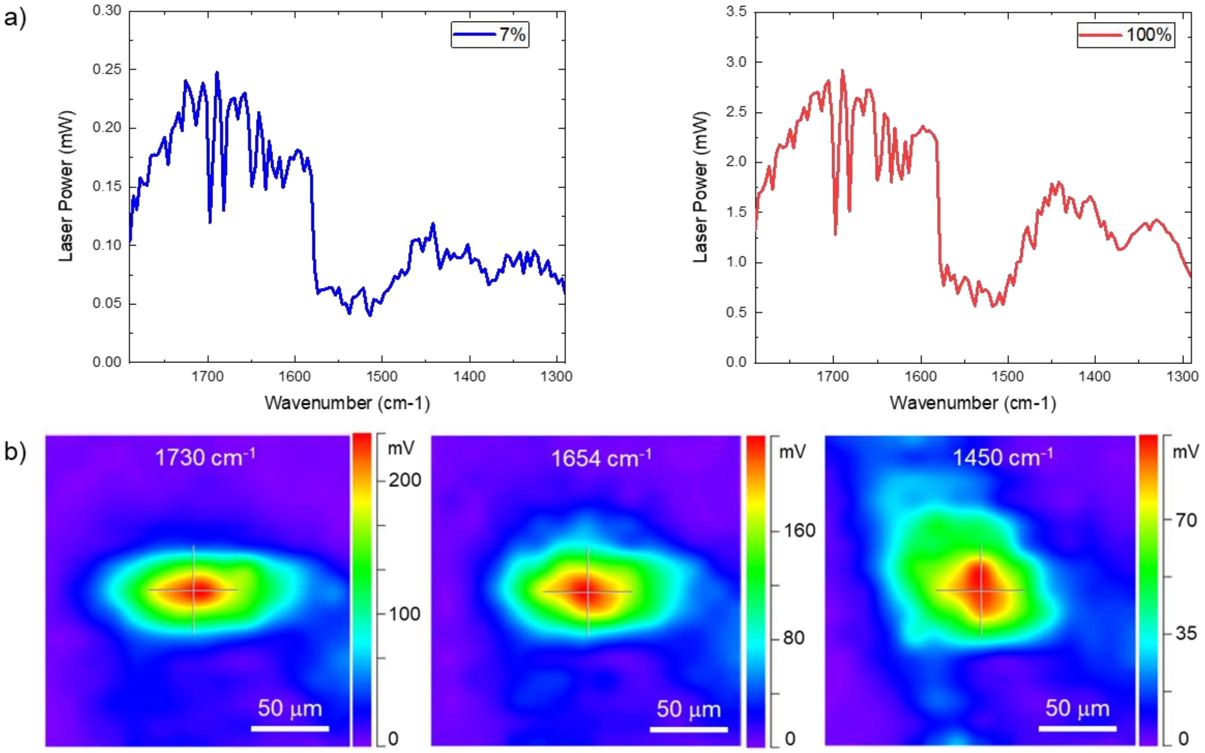

Supplementary Figure 1. QCL laser power and beam size. (a) Profile at 7% and 100% (100 ns) of laser maximum power after introduction of the mesh filter. The laser profile shows lower power in the regions of amide bands I and II, thus leading to lower signal to noise ratio when measuring a single protein. (b) The beam size of the QCL laser at the probe, in contact with the substrate, has a FWHM of 60 ± 10 µm and a $1/e^2$ of 60 ± 10 µm in the spectral range between 1800-1400 cm^{-1}, in good agreement with previous reports.[2] The beam size is measured at a laser power of ~0.2 mW (1730 cm^{-1}), ~0.22 mW (1654 cm^{-1}) and ~0.1 mW (1450 cm^{-1}) with laser pulse of 100 ns. In order to operate in linear regime of AFM-IR thermomechanical response (**Figure 1**), we could calculate a maximum allowed laser intensity $I = 4 \cdot 10^{-5}$ $mW \cdot \mu m^{-2}$. Source data are provided as a Source Data file.

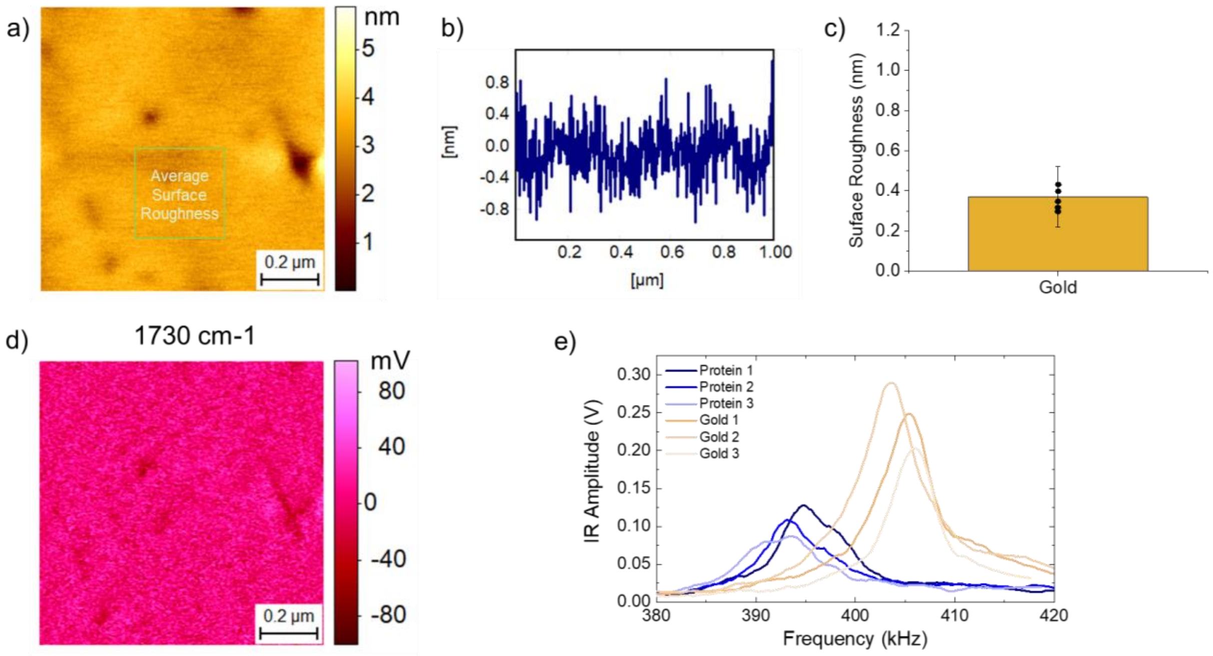

Supplementary Figure 2. Characterisation of the template-stripped gold substrate. (a) AFM morphology map acquired by the AFM-IR system on commercial gold substrate (Platypus technologies, USA) with nominal root mean squared roughness (RMS) of 0.36 nm. (b) Example of the roughness of a profile line of the map. (c) The measurement of the root mean square (green square, RMS) roughness of the surface in 5 different surface areas (average + (s.d. + ~0.1 nm electrical noise) is in excellent agreement with the nominal value. (d) AFM-IR map of IR absorption on gold shows a uniform response at 1730 cm^{-1}. (e) IR amplitude as a function of frequency pulse for signal on gold and on a thyroglobulin molecule at a laser power of 0.22 mW and at 1655 cm^{-1}. Source data are provided as a Source Data file.

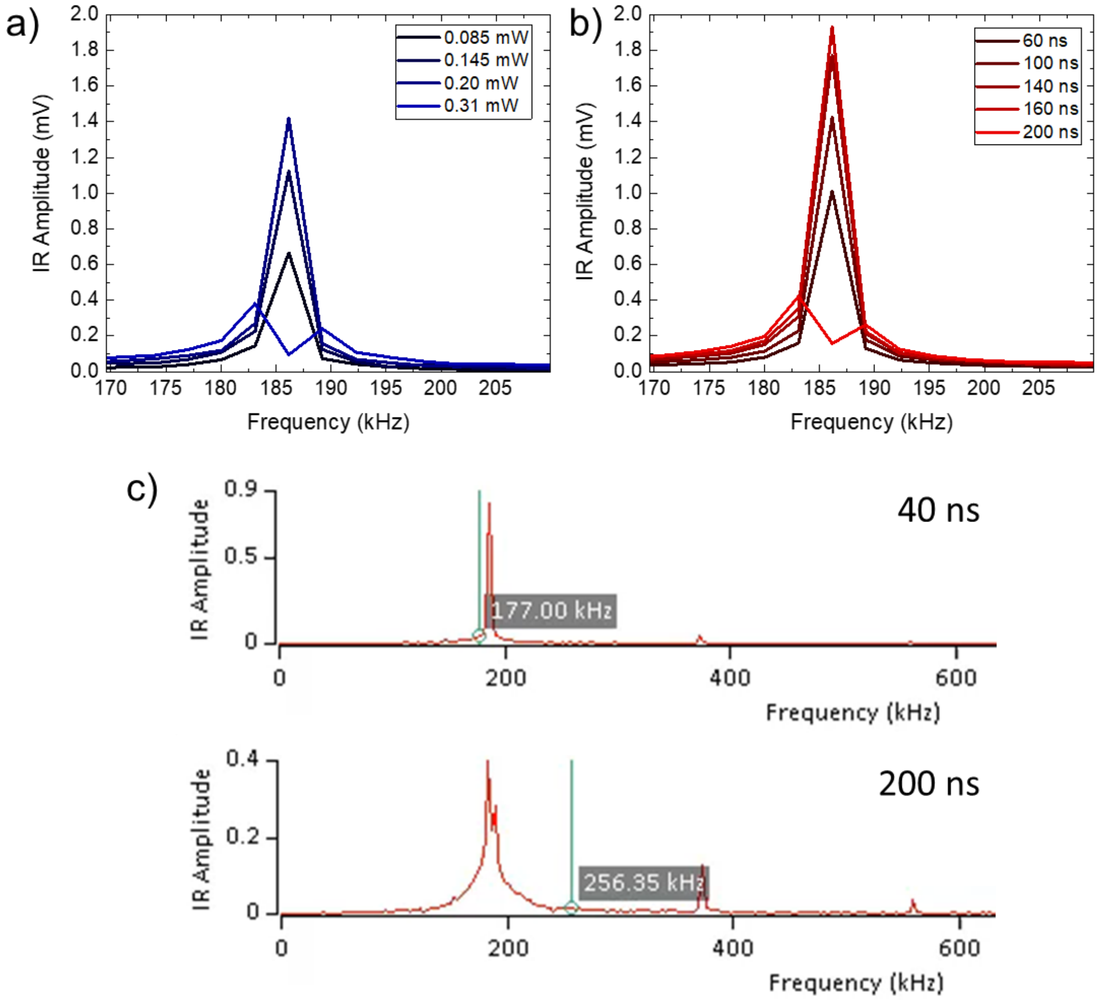

Supplementary Figure 3. Stability of AFM-IR signal as a function of power and pulse width of the laser. (a) IR Amplitude signal at 1730 cm^{-1} as a function of laser power showing instability of the cantilever response already at 0.31 mW and pulse width of 100 ns. (b) IR Amplitude as a function of pulse width of the laser at 1730 cm^{-1}, at a constant power of 0.20 mW, showing instability of the cantilever response already at 200 ns. (c) Frames of the Supplementary Movies 1-2 of the cantilever response at 40 ns and 200 ns with a laser power of 0.2 mW.

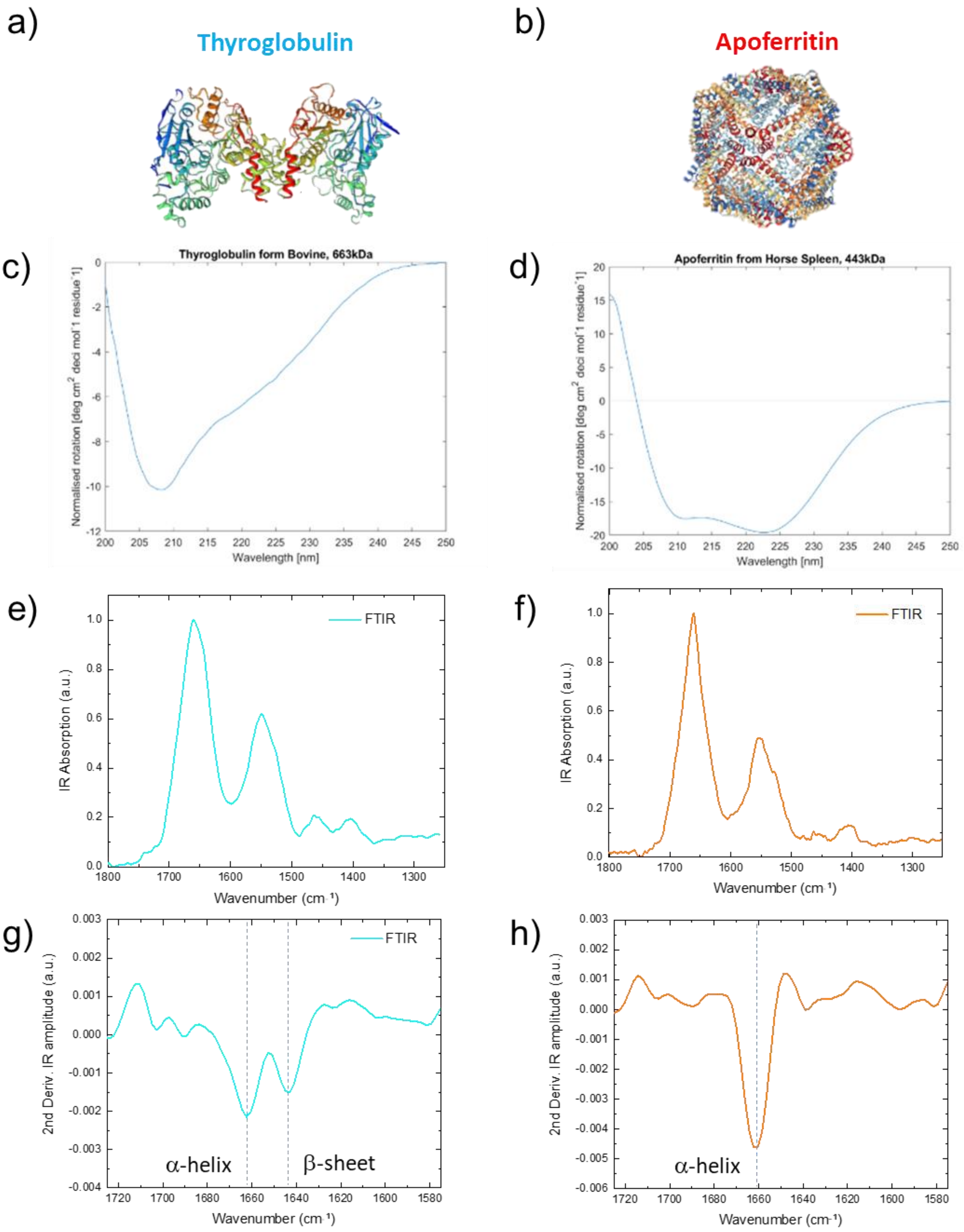

Supplementary Figure 4. Structures of thyroglobulin and apoferritin. Structures of: (a) thyroglobulin[3] and (b) apoferritin. CD measurements of: (c) thyroglobulin indicating presence of random coil and β-sheets, and (d) apoferritin indicating α-helical conformation. Bulk FTIR spectra of: (e) thyroglobulin and f) apoferritin, and (g,h) their 2nd derivatives, which show the presence of major contributions from intramolecular β-sheet and α-helix. Source data are provided as a Source Data file.

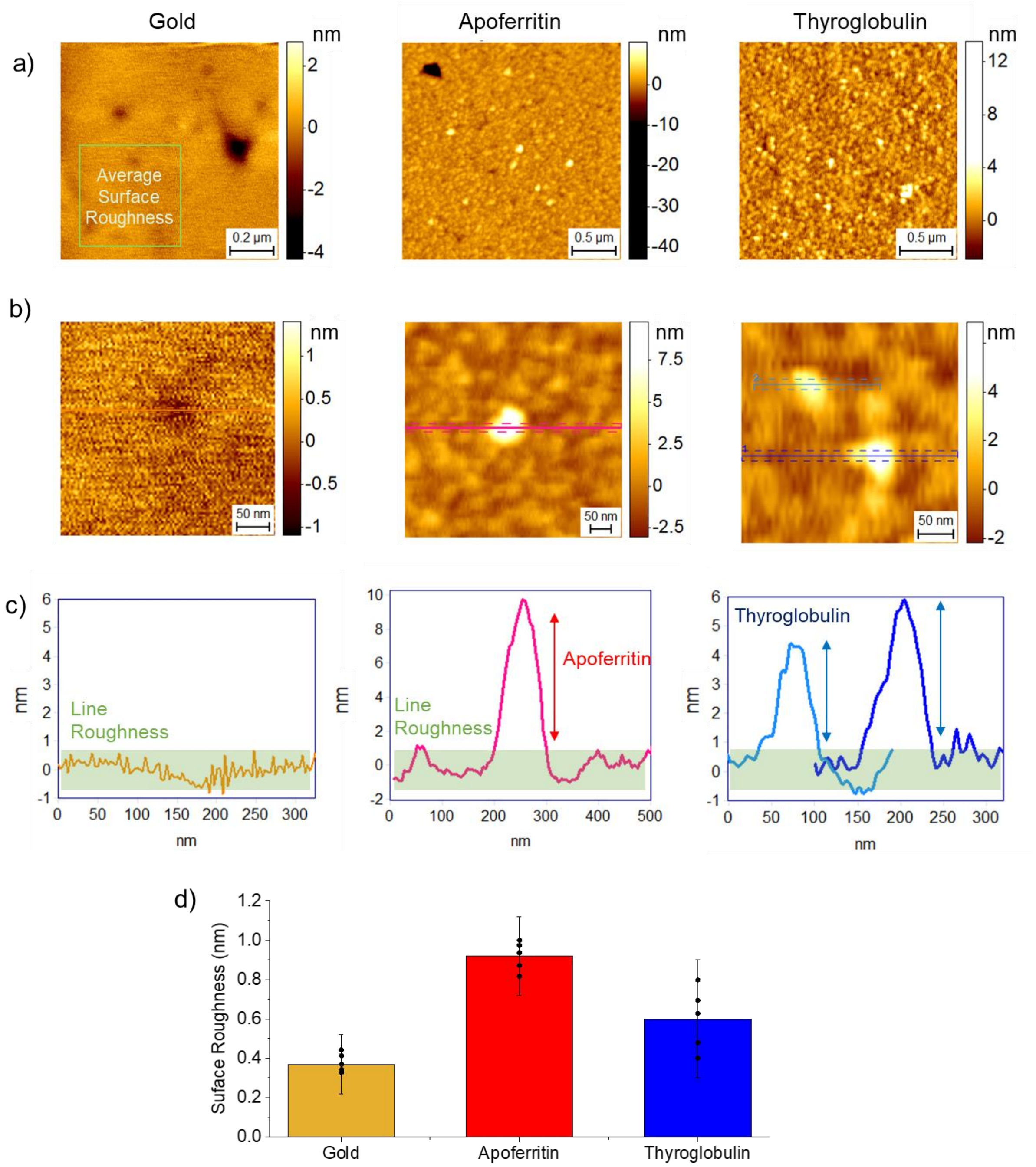

Supplementary Figure 5. Comparison of the roughness of the gold substrate and protein detection before and after protein deposition. (a-b) AFM morphology and detail of the freshly stripped gold substrate before and after protein deposition for AFM-IR analysis. (c) Cross-sectional profiles of the AFM maps (coulored orizontal lines). Even if protein deposition causes increase of surface roughness, single proteins are significantly discriminated above the roughness of the surface. (d) Comparison of roughness measurements before and after the deposition of the proteins on gold. In particular, apoferritin is in a 50% glycerol solution, causing an increase of the surface roughness after deposition. The error is the SD from the average of the roughness of 5 indipendent regions in the map plus the electrical noise of the AFM system of ~0.1 nm. Source data are provided as a Source Data file.

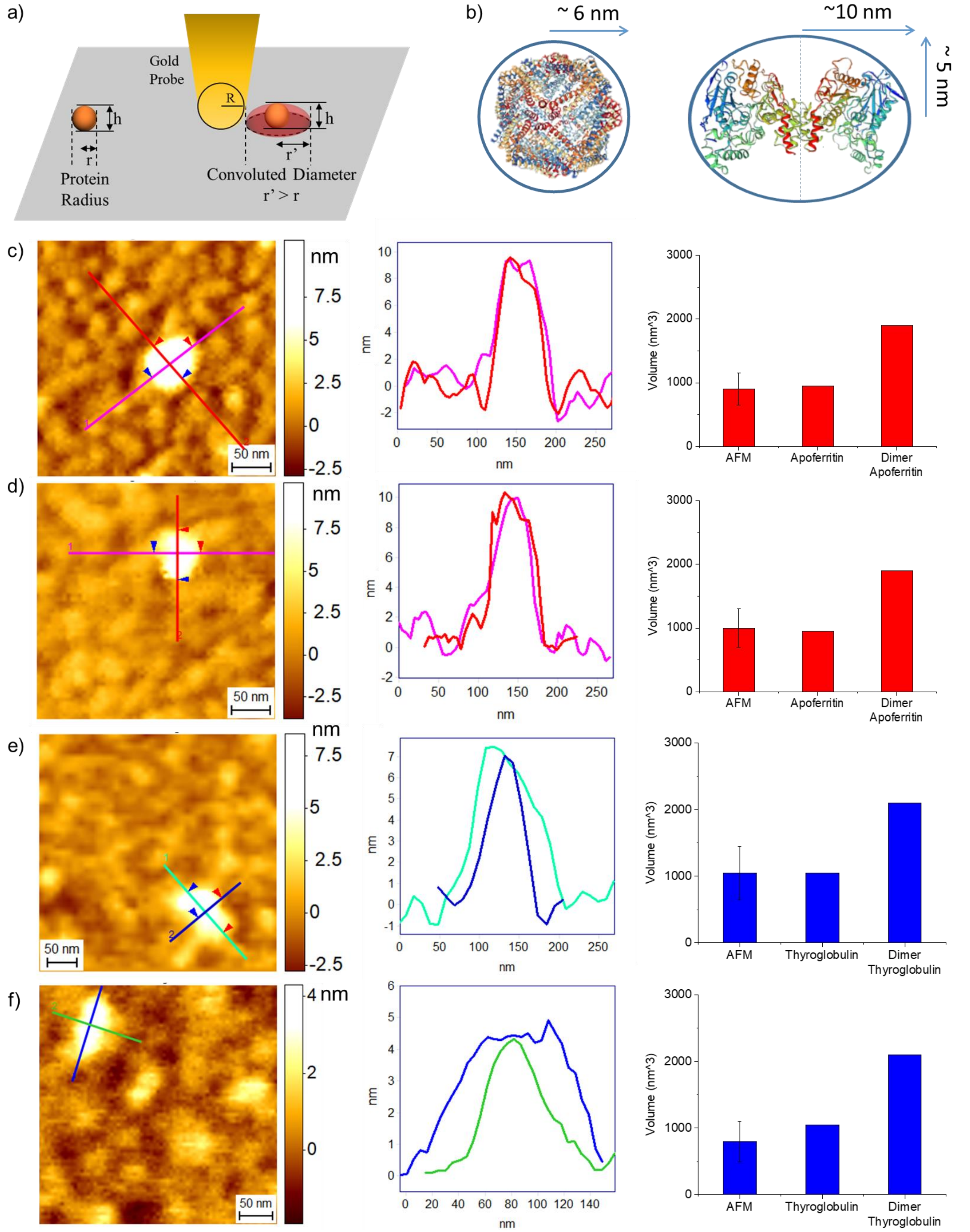

Supplementary Figure 6. Protein species measured by AFM-IR are single apoferritin and thyroglobulin molecules. (a) Convolution effect in AFM-IR causes an overestimation of the lateral dimensions and of the volume of the protein.[6] (b) Hydrodynamic radii of apoferritin (~6 nm) and thyroglobulin (~8 nm) and their crystal structures were used to estimate their volumes.[3,4] (c-f) AFM maps and cross sectional profile lines (left), measurement of the 3D cross-sectional dimensions (middle) and the deconvolution of the lateral dimensions of each single protein (right) was used to calculated the deconvoluted volume measured by AFM for (c-d, right) apoferritin and (e-f, right) thyroglobulin. The deconvoluted volume measured by AFM-IR is consistent with the volume of a single monomeric protein in both cases, in addition the measure volume is significantly smaller than the volume of two protein clustered (termed dimer). Thus the species observed in the morphology maps can be only single monomeric proteins. The error on the volume of each single protein is calculated by varying the nominal radius of the tip between R=30-40 nm. Source data are provided as a Source Data file.

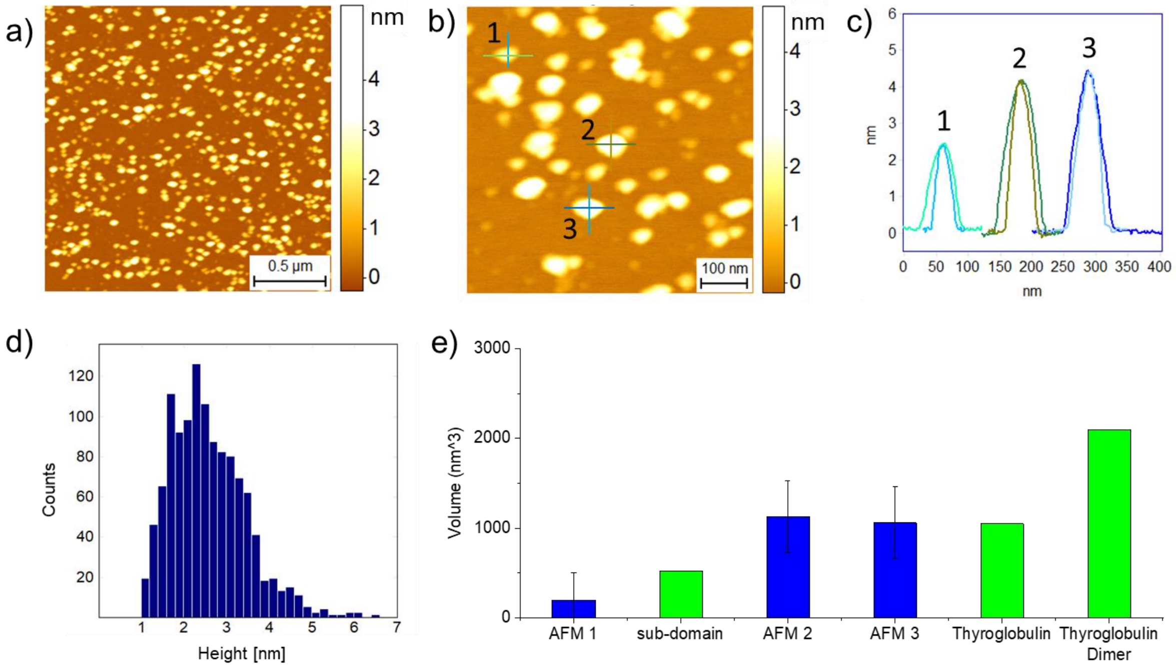

Supplementary Figure 7. Measurement of the volume of a thyroglobulin protein molecule by high-resolution AFM on mica substrate. (a-b) AFM morphology map and detail of thyroglobulin with the lines indicating where the cross-sectional profiles were acquired. (c) Cross-sectional dimensions of three selected proteins and (d) Statistical analysis of the cross-sectional height of the protein species. The lateral cross-sectional profiles are sharper since we used tip with smaller radius (~8 nm) than gold coated probes for AFM-IR (~30 nm). (e) The analysis of the deconvoluted volume on three example proteins by high-resolution AFM is in excellent agreement with the results obtained by AFM-IR (**Fig. 2** and **Supplementary Fig. S6**). The deposition on mica, similarly to gold deposition, shows the presence on the surface of monomeric thyroglobulin as well as species with smaller volume, similar to the volume of the thyroglobulin subdomain alone. The error on the volume of each single protein is calculated by varying the nominal radius of the tip for deconvolution between R=8-12 nm. Source data are provided as a Source Data file.

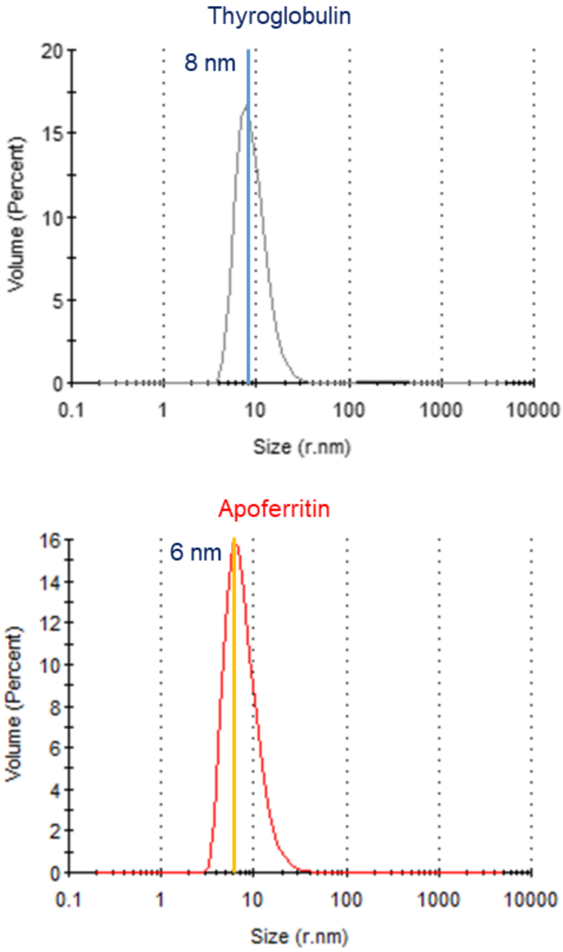

Supplementary Figure 8. Dynamic light scattering of thyroglobulin and apoferritin. Both proteins have a broad distribution of hydrodynamics radii varying approximately between 4-10 nm and peak (a) at ~8 nm for thyroglobulin and (b) at ~6 nm for apoferritin. The measurements demonstrate the monomeric state of the protein samples and they are in excellent agreement with previously reported data. Furthermore, these measurements confirm that the monomeric state in solution is at equilibrium with smaller sub-protein species as observed by AFM and AFM-IR. Source data are provided as a Source Data file.

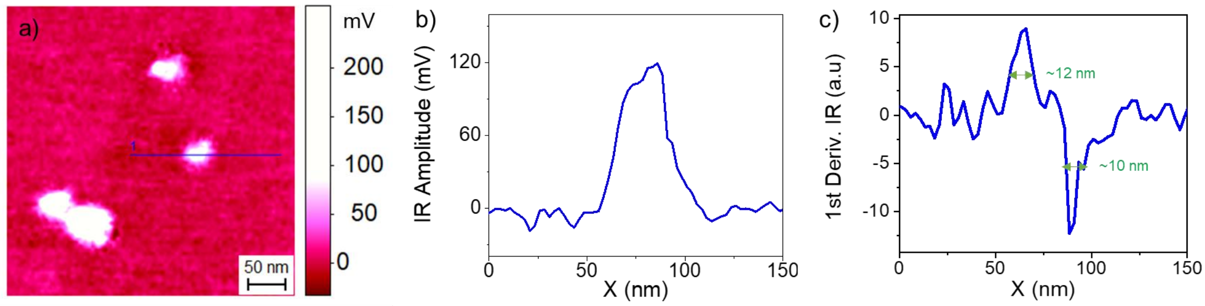

Supplementary Figure 9. AFM-IR Lateral Spatial Resolution. (a) AFM-IR infrared absorption map and (b) horizontal cross-section of the IR amplitude response. (c) To estimate quantitatively and more accurately the spatial resolution, we performed a first derivative of the section, we fitted we measured its full width at half maximum (FWHM). This quantity is intimately related to the sharpness of the transition and to our spatial resolution, which was in the best case ~10 ± 2.5 nm, where the error is determined by the pixel size on the X direction.

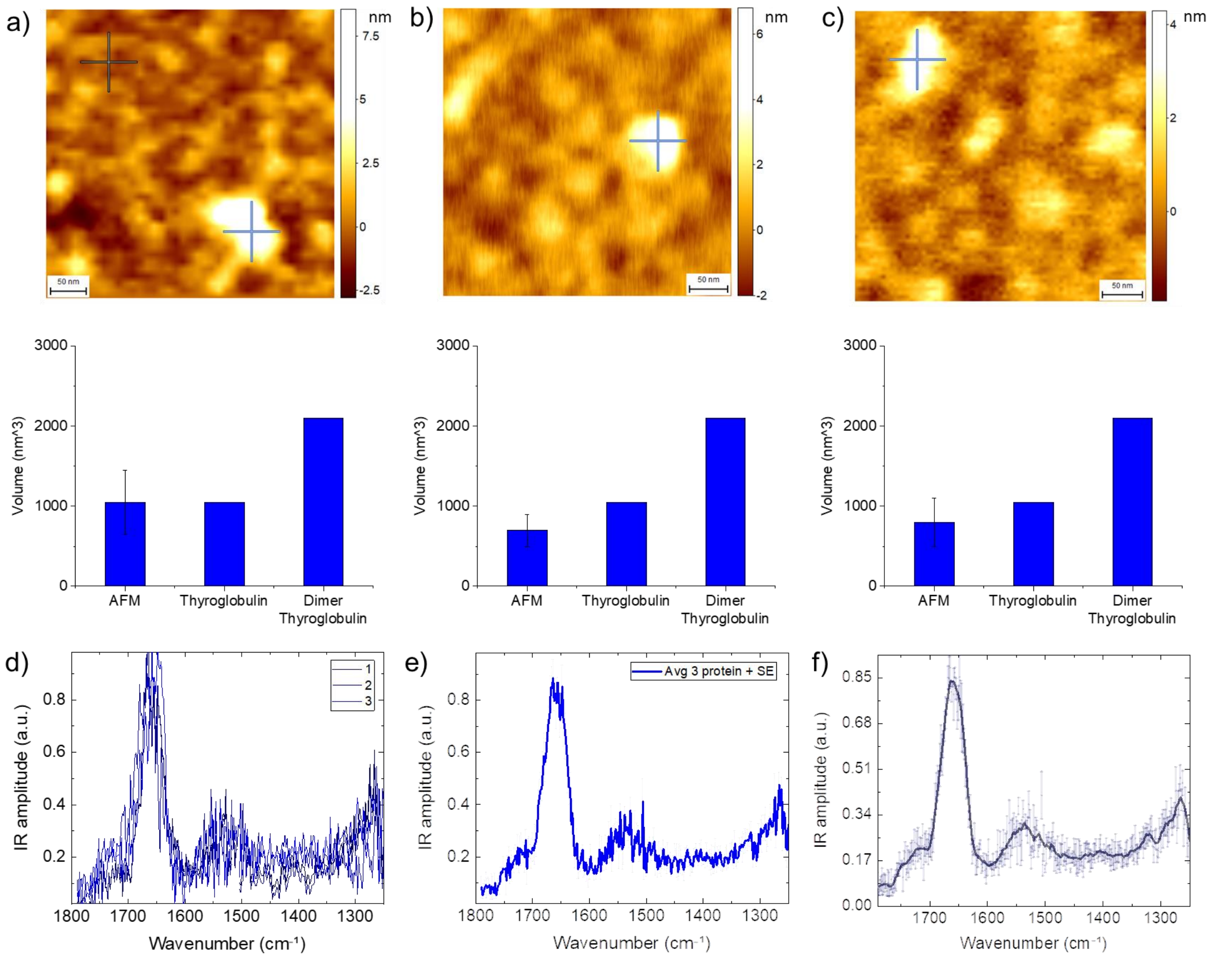

Supplementary Figure 10. Infrared absorption spectroscopy of individual thyroglobulin molecules. (a-c) AFM morphology maps of individual thyroglobulin molecules and their deconvoluted volume. (d) Spectra acquired on the proteins (blue crosses) and on the substrate (black cross), (e) their average with standard error (s.e.) and the smoothed spectrum by Savitzky-Golay filter (2nd order, 13 points). The error on the deconvoluted volume of each single protein measured is calculated by varying the nominal radius of the tip between R=30-40 nm. Source data are provided as a Source Data file.

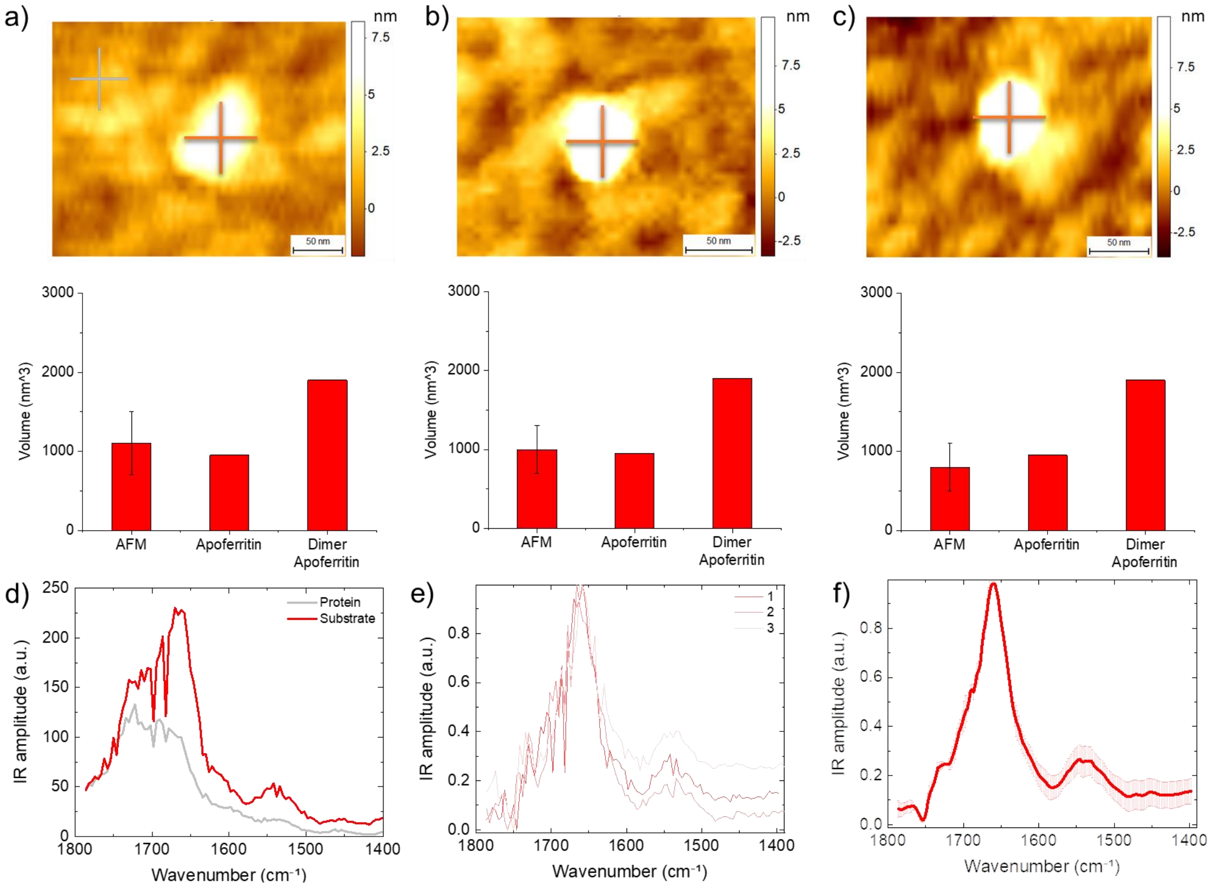

Supplementary Figure 11. Infrared absorption spectroscopy of individual apoferritin molecules. (a-c) AFM morphology maps of apoferritin and their deconvoluted volume. (d) Example of spectra acquired on the protein (red crosses) and on the substrate (grey cross). (e) Subtracted spectra of the proteins, and (f) their average with standard error (s.e.) smoothed by Savitzky-Golay filter (2nd order, 13 points). The error on the deconvoluted volume of each single protein measured is calculated by varying the nominal radius of the tip between R=30-40 nm. Source data are provided as a Source Data file.

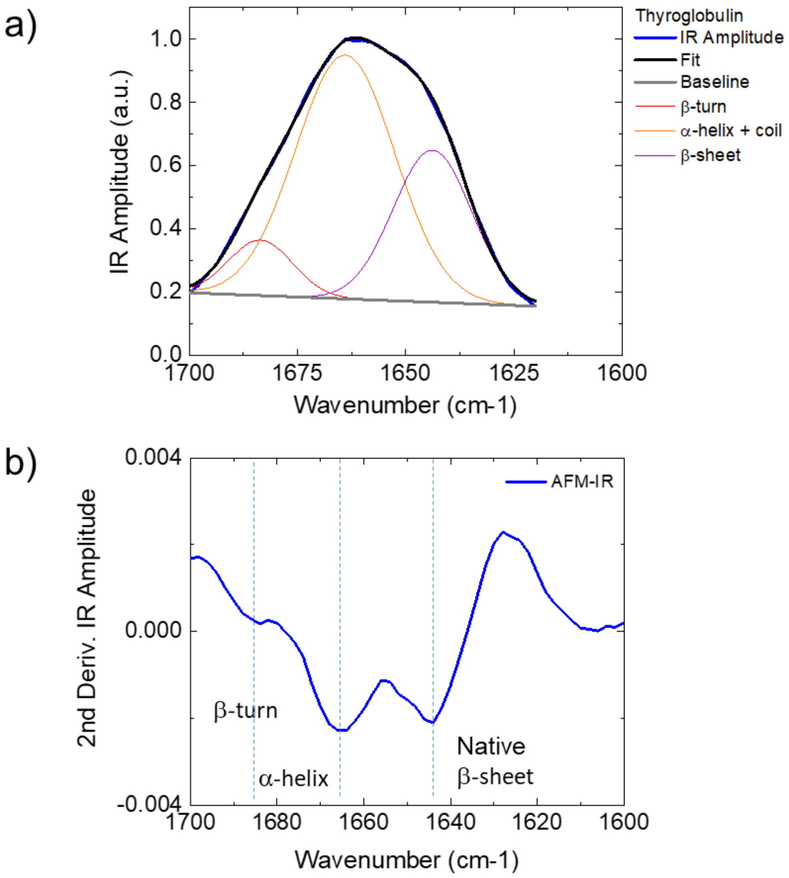

Supplementary Figure 12. Secondary structure evaluation method. We first considered the baselined amide band I of the protein of interest (a), and calculated its second derivative (b), which we used to de-convolve the band and finding the number of structural contributions and their chemical position. We fix these parameters (number of contribution and wavenumber) to fit the measured spectrum of the amide band I. The relative area of each structural contribution over the whole area of the peak is used to calculate the final percentage of the structural contributions, which has been assigned as widely accepted in literature and in previously published studies.[7,8]

SUPPLEMENTARY REFERENCES

1 Jin, M., Lu, F. & Belkin, M. A. High-sensitivity infrared vibrational nanospectroscopy in water. *Light Sci Appl* **6**, e17096, (2017).

2 Lu, F., Jin, M. & Belkin, M. A. Tip-enhanced infrared nanospectroscopy via molecular expansion force detection. *Nat Photon* **8**, 307-312, (2014).

3 Gunčar, G., Pungerčič, G., Klemenčič, I., Turk, V. & Turk, D. Crystal structure of MHC class II-associated p41 Ii fragment bound to cathepsin L reveals the structural basis for differentiation between cathepsins L and S. *The EMBO Journal* **18**, 793-803, (1999).

4 Kashanian, S., Abasi Tarighat, F., Rafipour, R. & Abbasi-Tarighat, M. Biomimetic synthesis and characterization of cobalt nanoparticles using apoferritin, and investigation of direct electron transfer of Co(NPs)-ferritin at modified glassy carbon electrode to design a novel nanobiosensor. *Mol Biol Rep* **39**, 8793-8802, (2012).

5 Giliberti, V. *et al.* Tip-Enhanced Infrared Difference-Nanospectroscopy of the Proton Pump Activity of Bacteriorhodopsin in Single Purple Membrane Patches. *Nano Letters* **19**, 3104-3114, (2019).

6 Ruggeri, F. S., Sneideris, T., Vendruscolo, M. & Knowles, T. P. J. Atomic Force Microscopy for Single Molecule Characterisation of Protein Aggregation. *Arch Biochem Biophys* **664**, 134-148, (2019).

7 Ruggeri, F. S. *et al.* Infrared Nanospectroscopy Characterization of Oligomeric and Fibrillar Aggregates during Amyloid Formation. *Nat Commun* **6**, 7831 (2015).

8 Shimanovich, U. *et al.* Silk Micrococoons for Protein Stabilisation and Molecular Encapsulation. *Nat Commun* **8**, 15902 (2017).
