# Supplementary material for: Single molecule secondary structure determination of proteins through infrared absorption nanospectroscopy
Source: Nat Commun. 2020 Jun 10;11:2945. doi: 10.1038/s41467-020-16728-1 (PMC7287102; doi:10.1038/s41467-020-16728-1)
Supplement: Supplementary file 3 — Description of Additional Supplementary Information [file 41467_2020_16728_MOESM3_ESM.pdf]

Description of Additional Supplementary Files

File Name: Supplementary Movie 1
Description: Thermomechanical response of the AFM-IR system as a function of time at a laser pulse of 40 ns and power of 0.2 mW. The IR amplitude of the AFM-IR signal is not saturated and stable.

File Name: Supplementary Movie 2
Description: Thermomechanical response of the AFM-IR system as a function of time at a laser pulse of 40 ns and power of 0.2 mW. The IR amplitude of the AFM-IR signal is not saturated but highly unstable.
